# Experience of discrimination during COVID-19 pandemic: the impact of public health measures and psychological distress among refugees and other migrants in Europe

**DOI:** 10.1186/s12889-022-13370-y

**Published:** 2022-05-11

**Authors:** Mattia Marchi, Federica Maria Magarini, Antonio Chiarenza, Gian Maria Galeazzi, Virginia Paloma, Rocío Garrido, Elisabeth Ioannidi, Katerina Vassilikou, Margarida Gaspar de Matos, Tania Gaspar, Fabio Botelho Guedes, Nina Langer Primdahl, Morten Skovdal, Rebecca Murphy, Natalie Durbeej, Fatumo Osman, Charles Watters, Maria van den Muijsenbergh, Gesine Sturm, Rachid Oulahal, Beatriz Padilla, Sara Willems, Eva Spiritus-Beerden, An Verelst, Ilse Derluyn

**Affiliations:** 1 Department of Biomedical, Metabolic and Neural Sciences, University of Modena and Reggio Emilia, Via Giuseppe Campi, 287 –, 41125 Modena, Italy; 2 Dipartimento di Salute Mentale e Dipendenze Patologiche, Azienda USL-IRCCS di Reggio Emilia, Via Giovanni Amendola 2 –, 42122 Reggio Emilia, Italy; 3 Department of Social Psychology, Universidad de Sevilla, 41018 Seville, Spain; 4 Research Center for Greek Society, Academy of Athens, 15126 Athens, Greece; 5 Institute of Environmental Health/ISAMB, University of Lisbon, Lisbon, Portugal; 6 Department of Public Health, University of Copenhagen, 1014 Copenhagen, Denmark; 7 Department of Psychology, Maynooth University, W23 F2K8, Co. Kildare Maynooth, Ireland; 8 Department of Child Health and Parenting, Uppsala University, 75236 Uppsala, Sweden; 9 School of Health and Welfare, Dalarna University, Högskolegatan 2, 79188 Falun, Sweden; 10 Department of School of Education and Social Work, University of Sussex, Sussex, UK; 11 Department of Primary and Community Care, Radboud University, 6500 HB Nijmegen, The Netherlands; 12 LCPI Laboratory, EA-4591, Department Clinique du Sujet, University of Toulouse 2, 31058 Toulouse, France; 13 La Reunion University FR, DIRE research center, French Collaborative Institute on Migration, CS 92003, 15 Av. René Cassin, Saint-Denis, Cedex 9 97400 Réunion; 14 Department of Sociology, University of South Florida, Tampa, FL 33620 USA; 15 Department of Public Health and Primary Care, Quality and Safety Ghent, Ghent University, 9000 Ghent, Belgium; 16 Department of Social Work and Social Pedagogy, Ghent University, 9000 Ghent, Belgium

**Keywords:** COVID-19, Migrants, Social stigma, Mental health, Public health

## Abstract

**Background:**

The COVID-19 pandemic has had a disproportionately hard impact on refugees and other migrants who are often exposed to the virus with limited means to protect themselves. We tested the hypothesis that during the COVID-19 pandemic, refugees and other migrants have suffered a negative impact on mental health and have been unjustly discriminated for spreading the disease in Europe (data collection from April to November 2020).

**Methods:**

Participants in the ApartTogether Survey (*N =* 8297, after listwise deletion of missing items final *N =* 3940) provided data regarding to their difficulties to adhere to preventive recommendations against COVID-19 infection (CARE), self-perceived stigmatization (SS), and psychological distress (PD). Structural Equation Modeling was used to investigate PD as a mediator in the pathway linking CARE to SS, while adjusting for the housing and residence status. To improve confidence in the findings, single hold-out sample cross-validation was performed using a train/test split ratio of 0.8/0.2.

**Results:**

In the exploratory set (*N =* 3159) SS was associated with both CARE (B = 0.200, *p <* 0.001) and PD (B = 0.455, *p <* 0.001). Moreover, PD was also associated with CARE (B = 0.094, *p* = 0.001) and mediated the effect of CARE on SS (proportion mediated = 17.7%, p = 0.001). The results were successfully replicated in the confirmation set (*N =* 781; total effect = 0.417, *p <* 0.001; proportion mediated = 29.7%, *p <* 0.001). Follow-up analyses also found evidence for an opposite effect (i.e., from SS to CARE, B = 0.132; *p <* 0.001), suggesting that there might be a vicious circle between the self-perceived stigmatization and the access to health care and the use of preventive measures against COVID-19 infection.

**Conclusions:**

Refugees and other migrants who had more difficulties in accessing health care and preventive measures against COVID-19 infection experienced worse mental health and increased discrimination. These negative effects appeared to be stronger for those with more insecure housing and residence status, highlighting from one side the specific risk of insecure housing in the impact of COVID-19 upon mental health and infection protection, and for another side the need to proper housing as a strategy to prevent both COVID-19 and mental distress.

**Supplementary Information:**

The online version contains supplementary material available at 10.1186/s12889-022-13370-y.

## Introduction

The COVID-19 pandemic has had a disproportionately hard impact on refugees and other migrants who are often exposed to the virus with limited means to protect themselves. Basic public health measures, such as social distancing, maintaining hand hygiene, and self-isolation are not possible or extremely difficult to implement in situations where many people are in close contact and gather in large groups, such as in refugee camps and the overcrowded housing of many other immigrants with low income or insecure status. Such conditions may have a considerable impact also on the mental health, with individual and public health implications [1, 2].

Previous research has identified migrants’ barriers in accessing both physical and mental health care, which likely worsened during the pandemic [3, 4]. These may be summarized within three groups: (1) cultural barriers, including mental health stigma and knowledge of dominant models of health; (2) structural barriers, including scarce culturally and linguistically accessible information, unstable accommodation, and financial strain; and (3) barriers specific to the migrant experience, including the consequences of postponement of decisions on their legal status, resettlements, or border closures. In spite of worldwide calls to action for inclusion of refugees and other migrants in the pandemic response [5, 6], there have been reports of increased stigmatization and xenophobia across the world since the onset of the COVID-19 pandemic [7, 8]. Skovdal et al. [9] argue that this may be explained by how risk communication about COVID-19 constructs new social norms about how to act and behave in public, which may inadvertently contribute to a blaming and shaming of those who are unable to comply. This disproportionately affects already stigmatized groups, such as refugees and other migrants who are typically discriminated against for spreading disease to host populations, despite little evidence to support this [10]. Actually, the transmission is more likely within refugee and migrant populations, e.g., due to crowded living conditions and scarce access to basic sanitation.

Previous studies have documented the role of racism in health inequalities, defining race as a “pathogenic factor” for both physical and mental illness [11, 12]. Compelling models on racial health disparities suggest that racism negatively impacts both individual wellbeing and health care utilization in many ways [13]. The manifestations of the phenomenon are varied, from institutional racism (e.g., in judicial or educational systems) to interpersonal racism (e.g., stigma, social exclusion), also within health care services when immigrant users “express disappointment with services that dismiss their concerns and fail to attend to their priorities” [14]. The perception of being discriminated against and excluded from social interactions has been linked with both increased psychological distress and worse physical health [15–18]. In addition, studies on mediating factors suggested that the negative effect of stigmatization on physical health might be in part exerted through increased psychological distress [19–21]. In other infectious disease epidemics, social stigma and self-perceived discrimination have been found to amplify the dangers related to infection and engagement with preventative measures, treatment, and care. For example, people who perceived more HIV-related stigma had a two-fold probability of delayed treatment seeking and higher viral load at first healthcare contact [22–24]. Qualitative studies in the field of HIV continue to find different forms of stigma to negatively affect engagement with medical care [25]. The situation is even more alarming among undocumented and migrant populations, where added to disease-related stigma, people might fear losing social support or not being resettled – compounded by experiences of prejudice and discrimination linked to race and their migrant or refugee status [26]. Such conditions may create a vicious circle, negatively impacting mental health, psychological self-regulation, and treatment adherence – all of which carry important public health implications. According to the theory of Social Stigma [27, 28], in a prejudice-based context, the victims of racism are not passive actors of that circumstance, instead, they actively face the perception of being the object of discrimination, employing different coping strategies affecting their social interactions and the overall social inclusion process. In the context of COVID-19, an effect of social stigma and self-perceived discrimination, could be that refugees and other migrants are afraid of seeking treatment or disclosing symptoms leading to risks to wider public health outcomes, including for host populations [29].

Using the European data of the ApartTogether Survey [30], we aim to better understand the connection between the difficulties to adhere to preventive recommendations against COVID-19 infection (CARE) and self-perceived stigmatization (SS), focusing on the role of psychological distress (PD). Specifically, we hypothesize that PD might be a mediator in the relationship between CARE and SS, and that there might be a bidirectional relationship between the self-perceived stigmatization and the access to health care and the use of preventive measures against COVID-19 infection.

## Methods

### Study design and population

The plight of refugees and other migrants during the (ongoing) COVID-19 crisis has been highlighted in the media, and by online stories, blogs, reports, and posts, often with regional or national focus and not reported by refugees and migrants themselves. The ApartTogether is a perception-based survey, prompted by the WHO Global Programme for Health and Migration in collaboration with a large European consortium of academics, and was the first inquiry into the social impact of the COVID-19 pandemic on refugees and other migrants globally [30]. The survey gave refugees and other migrants an online platform to self-report the perceived impact of COVID-19 on their lives including the preventive measures recommended. It is not a systematic survey of the incidence of COVID-19 among refugees and migrants nor an assessment of the prevalence of symptoms or immunity, rather it aims to give refugees and other migrants a voice to better understand their situation and specific difficulties they face during the COVID-19 pandemic, to provide information for policy makers and inform the health systems on preparedness and inclusiveness [31].

Data were collected from the ApartTogether online global survey, which ran from April 2020 until November 2020. The current study used the European data from 46 different countries and mainly focused on three different parts of the ApartTogether Survey, consisting of: difficulties to adhere to preventive recommendations against COVID-19 infection (CARE), experience of stigma and self-perceived discrimination (SS), and mental health (i.e., the level of self-reported psychological distress [PD]). In total, *N =* 8297 refugees and migrants hosted in Europe entered the survey, however given the participants could stop the survey at any time, not everyone completed all the items, thus the number of full entries used in this study was *N =* 3940.

### Study questionnaire

Participants completed questions about sociodemographic characteristics, including age, gender (i.e., male, female, non-binary), education, housing situation (i.e., living in a house or apartment, asylum center for asylum seekers - meant as someone who is seeking international protection but whose claim for refugee status has not yet been determined - refugee camp for refugees waiting for resettlement, or on the street), residence status (i.e., citizen, permanent documents, temporary documents, no documents), and working situation (i.e., student, employed, unemployed since the corona-crisis, or already unemployed before corona-crisis).

To investigate the difficulties in adhering to preventive recommendations against COVID-19 infection (i.e., the CARE latent construct), participants were asked how frequently they engage in handwashing, keeping physical distance from others, covering nose and mouth in public, avoiding using public transport and going out of the house. The possible answers were structured on a 4-levels Likert-type scale consisting of: “No, because I am not able”, “No, because I don’t want”, “Yes, sometimes”, and “Yes, all the time”. In order to focus on the overall utilization of precautions, the answers were coded as “Never” (i.e., including both “I don’t want” and “I am not able” replies – yet description of all possible answers is available in the Supplement), “Sometimes”, “Always”. In addition, participants were asked if they would contact a doctor if they or a person close to them would develop COVID-19 symptoms. Further, participants reported changes in their mental health from COVID-19 pandemic (i.e., the PD latent construct) through indicating how often they experienced feelings of depression, anxiety, loneliness, anger, irritation, hopelessness, unpleasant reminders of past traumatic experiences, physical reactions to stress, sleeping problems, and substance use, such as alcohol and drugs. Responses were collected over a Likert-type scale on 4-levels: “Never”, “Seldom”, “Sometimes”, “Always”. Alongside, they reported also on a binomial “worse-scale” (as “Yes” or “No”) if these mental health problems worsened in respect to before COVID-19. To maximize the sample size, we primarily focused on the “frequency” questions which had many more entries (e.g., if a person replied “never” in the frequency question, in all probability left the “worse-scale” empty).

Finally, to measure the experiences of discrimination of respondents (i.e., the SS latent construct), six items were investigated: being differently treated because of your origin, being called names because of your origin or religion, being avoided, other people being anxious of you, being unfairly treated by the police, being treated with kindness. Respondents were asked to indicate on a binomial scale, as “Yes” or “No”, whether they felt their treatment by others had worsen since the outbreak of the COVID-19.

### Statistical analysis

The analyses were conducted with structural equation modeling (SEM) using the *lavaan* [32], *tid*y*SEM* [33], and *SEMsens* [34] packages (versions: 0.6-10; 0.1.3.1; 1.0.1, respectively) in R (2021.09.2 + 382 “Ghost Orchid” Release for macOS) [35]. Missing data rates ranged from 14 to 47% and were listwise deleted, because multiple imputation was not possible for ordinal factors in the *lavaan* package, therefore the final N used in the analyses was 3940.

Previous to the SEM, confirmatory factor analysis was performed on the full set of complete data (*N =* 3940) to determine if the theorized measurement models have an acceptable fit for each latent construct (i.e., CARE, PD, and SS). Next, the total sample was randomly split into exploratory and confirmation sets using a split ratio of 0.8/0.2 (i.e., *N =* 3159 in the exploratory set, and *N =* 781 in the confirmation set). We performed a mediation analysis using diagonally weighted least squares (DWLS) estimator to test whether CARE exerts an effect on SS both directly and indirectly through PD. The bias-corrected bootstrap 95% confidence interval (95% CI) for the indirect effect was tested to be entirely above zero based on 5000 bootstrap samples applying the adjusted bootstrap percentile method (BCa). Moreover, we evaluated the model results’ sensitivity to an omitted confounder using the ant colony optimization algorithm (based on k = 100, and maximum iterations = 1000) to automatically search for sensitivity parameters that would lead to a change in the study conclusions.

The following fit indices were considered as indicating good fit: Root Means Square Error of Approximation (RMSEA) < 0.05 (< 0.08 indicates adequate fit); Standardized Root Means Square Residual (SRMR) < 0.08; Tucker–Lewis Index (TLI) and Comparative Fit Index (CFI) > 0.95 [36–39]. Cronbach’s alpha was used to test the reliability of the latent variables, deeming a value of 0.70 or higher as a satisfactory level of reliability [40].

The variance covariance matrix of the full set of data (*N =* 3940) is available as Supplementary Table 1.

## Results

### Description of the sample

The main characteristics of the sample analyzed in the SEM are displayed in Table 1 and detailed description of the study questionnaire answers is displayed in Supplementary Table 2.

**Table 1:** Characteristics of the sample that was analyzed in the SEM

Variable (***N =*** 3940)	Frequency (%)/Mean (SD)	Missing rates
Gender:		0
Male	1924 (48.8%)	
Female	2004 (50.9%)	
Non-binary	12 (0.3%)	
Age (years)	34.7 (10.3)	0
Education:		178 (4.5%)
No schooling	57 (1.4%)	
Primary school	171 (4.3%)	
Secondary school	852 (21.6%)	
Higher education	2682 (68.1%)	
Housing situation:		165 (4.2%)
House/Apartment	3500 (88.8%)	
Asylum center	155 (3.9%)	
Refugee camp	91 (2.3%)	
On the street	26 (0.7%)	
Residence status:		159 (4.0%)
Citizen in this country	860 (21.8%)	
Permanent documents	1140 (28.9%)	
Temporary documents	1524 (38.7%)	
No documents	255 (6.5%)	
Work situation:		617 (15.7%)
Student	691 (17.5%)	
Employed	1813 (46.0%)	
Unemployed since the corona-crisis	104 (2.6%)	
Already unemployed before corona-crisis	715 (18.1%)	
Have you tested positive for the COVID-19? *(current November 2020)* (No/Yes)	3528 (89.5%)/347 (8.8%)	65 (1.6%)

Abbreviations: *SD* Standard deviation, *COVID-19* Coronavirus disease 2019

Since there may be important country-specific differences in the access to health care and in the legal requirements in the COVID-19 response impacting participants’ adherence, although not fully investigable with inferential statistics due to the heterogeneous distribution of participants across the countries, we provided in the Supplementary Table 3 description of participants’ adherence to recommendations against COVID-19 by country.

### Latent variable estimation

The CARE dimension was built from the following questions: “What precautions are you taking to avoid transmitting the coronavirus?” and “In case I or one of your family members (or a person close to you) develops symptoms, I would contact a doctor or health care provider?”. The answers were homogeneously ordered so that more difficulties in adhering to preventive measures against the virus and in accessing care give rise to the CARE score. The Cronbach’s alpha for this latent variable was 0.73.

PD was estimated using the frequency answers (i.e., “No”, “Seldom”, “Sometimes”, “Always”) to the question: “Since the coronavirus and the corona measures, have you been bothered by the following problems?”, except for the item 2 (i.e., “worried”) that has been removed because collinear with item 3 (i.e., “anxious”). The answers were homogenously ordered, so that a more frequent engagement in that mental health experiences gives raise to the PD score. The Cronbach’s alpha for the latent variable PD was 0.91.

Finally, the following question investigated experience of SS: “Since the corona-crisis, do you feel differently treated by others than before?”. Reporting a worsening in the perceived discrimination than before the corona-crisis increases the SS score. The Cronbach’s alpha for SS was 0.95.

Supplementary Table 4 summarizes the contribution of the observed items to each latent variable.

### Mediation analysis in the exploratory set

From the mediation analysis conducted in the exploratory set (*N =* 3159), CARE influenced the level of SS both directly and indirectly through its effect on PD.

**Fig. 1:**
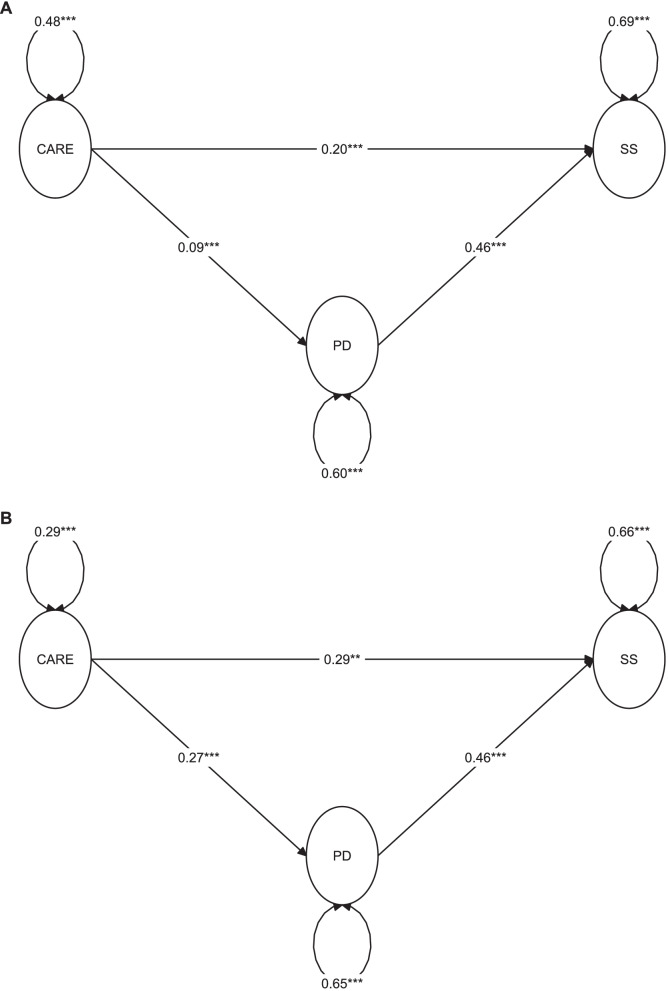
Mediation model in the exploratory set (Panel A:80%;*N =* 3159) and in the confirmation set (Panel B:20%;*N =* 781). The estimates reported are the unstandardized regression coefficients. *p < 0.05; **p < 0.01; ***p < 0.001. Abbreviations: CARE: difficulties to adhere to preventive recommendations against COVID-19 infection; PD: psychological distress; SS: self-perceived stigmatization

Table 2 and Fig. 1 (Panel A) show that subjects who less adhere to preventive recommendations against COVID-19 infection experienced more stigmatization (unstandardized regression beta [B] = 0.200; *p <* 0.001) and psychological distress (B = 0.094; *p* = 0.001). In addition, the people who reported higher PD also reported to perceive higher stigmatization (B = 0.455; *p <* 0.001). The bootstrap 95% CIs for the indirect effect through PD (B = 0.043) were entirely above zero (0.018 to 0.067), consistent with a significant contribution of CARE to SS, through PD.

**Table 2:** Results of the mediation model in the exploratory set (i.e., 80%; *N =* 3159 - above), and in the confirmation set (i.e., 20%; *N =* 781 - below)

Regressions	Unstandardized estimate (95% CI)	Standardized coefficient	***p***-value
*Exploratory set (80%)*			
**Direct effects**			
*Outcome model: SS (R*^*2*^ *= 0.182)*			
CARE	0.200 (0.126; 0.274)	0.151	< 0.001
PD	0.455 (0.397; 0.514)	0.387	< 0.001
*Mediator model: PD (R*^*2*^ *= 0.007)*			
CARE	0.094 (0.039; 0.148)	0.083	0.001
**Indirect effect (proportion mediated)**			
PD (17.7%)	0.043 (0.018; 0.067)	0.032	0.001
**Total effect**	0.243 (0.165; 0.321)	0.183	< 0.001
*Confirmation set (20%)*			
**Direct effects**			
*Outcome model: SS (R*^*2*^ *= 0.221)*			
CARE	0.293 (0.104; 0.482)	0.172	0.002
PD	0.457 (0.357; 0.558)	0.408	< 0.001
*Mediator model: PD (R*^*2*^ *= 0.032)*			
CARE	0.272 (0.120; 0.423)	0.179	< 0.001
**Indirect effect (proportion mediated)**			
PD (29.7%)	0.124 (0.056; 0.192)	0.073	< 0.001
**Total effect**	0.417 (0.215; 0.620)	0.245	< 0.001

Abbreviations: 95% CI: 95% bias-corrected bootstrap confidence interval, *SS* Self-perceived stigmatization, *CARE* Difficulties to adhere to preventive recommendations against COVID-19 infection, *PD* Psychological distress

The goodness of fit measures for this model were: CFI = 0.985; TLI = 0.983; RMSEA = 0.045; SRMR = 0.065, all indicating a good fit.

### Mediation analysis in the confirmation set

Table 2 and Fig. 1 (Panel B) show the mediation model in the confirmation set (*N =* 781). Both the direct (B = 0.239; *p* = 0.002) and the indirect effect - through PD - (B = 0.124; bootstrap 95% CI: 0.056 to 0.192) of CARE on SS were confirmed. The goodness of fit indices were indicating good fit: CFI = 0.988; TLI = 0.986; RMSEA = 0.042; SRMR = 0.078.

The relationship between PD and SS was fully retained also if building PD using the dichotomous “worse scale”, yet the association with CARE became not significant, likely due to loss of power (the missing rate for PD on the “worse scale” was 80%; data available on request). However, the PD “worse scale” was significantly associated with PD “frequency scale” (covariance = 0.487; r = 0.99; *p <* 0.001).

### Sensitivity analysis

To evaluate the sensitivity of our SEM to potential missing confounders, we used the Ant Colony Optimization algorithm [41–43]. Specifically, through that algorithm, we introduced for each path in the model a “phantom variable” which serves as a potential missing confounder. We relied on the changed *p*-values that are obtained with the introduction of the phantom variables. Note that NAs occur when there is no change in the *p*-value for any of the tested phantom variable path coefficients. The results of this analysis are displayed in Supplementary Table 5.

Following Kolenikov’s [44] recommendation that parameters with greater than 10% change can be considered sensitive to misspecification, we found that all the paths might be sensitive to potential confounders. However, when evaluating the change in *p-*values, we found that only the path from CARE to SS was no longer significant when the confounder is added. One implication of this result is that, at worse, the relation between CARE and SS is fully mediated by PD.

### Exploring other models

**Fig. 2:**
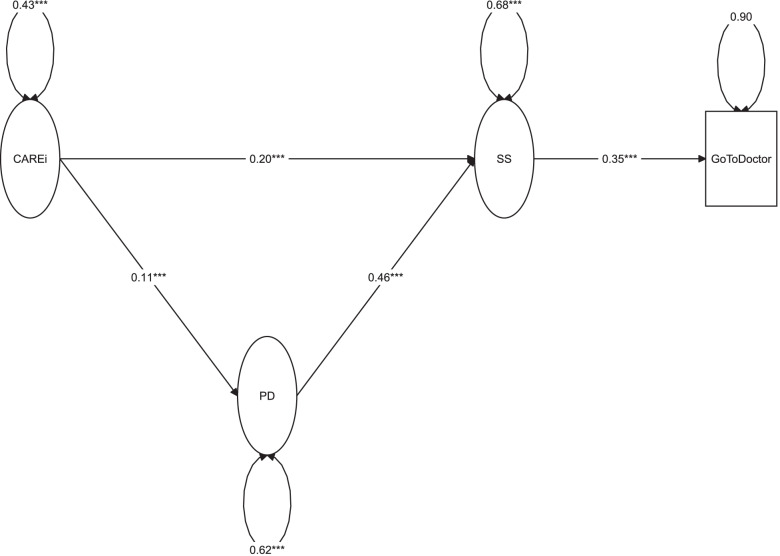
Multiple outcome mediation model in the full set. Legend: The estimates reported are the unstandardized regression coefficients. *p < 0.05; **p < 0.01; ***p < 0.001. Abbreviations: CAREi: difficulties to adhere to preventive recommendations against COVID-19 infection without question 19; PD: psychological distress; SS: self-perceived stigmatization

As a follow-up to the sensitivity test, two variations on the main model were assessed. To better distinguish the adherence to preventive measures against COVID-19 and the access to care (namely, question 19: “In case I or one of your family members develops symptoms, I would contact a doctor or health care provider?” [answer coded as flipped, “Yes/No”, thus higher score indicates that people would not go to a doctor in case of corona symptoms]), we fitted a first model where this question was removed from the latent variable CARE (new variable called CAREi), and modelled as the outcome of SS. So, the theorized model would add investigation of the impact of SS on the health care utilization. As can be seen in Table 3 and Fig. 2, our findings suggest that SS had a negative impact on the health care access, shown as an increased likelihood to not consult a doctor in case of COVID-19 symptoms (B = 0.353; *p <* 0.001). The Cronbach’s alpha for that newly conceptualized CAREi latent variable increased up to 0.88, and the fit indices were still indicating good fit: CFI = 0.985, TLI = 0.983, RMSEA = 0.045, SRMR = 0.062.

**Table 3:** Multiple outcome mediation model in the full set

Regressions	Unstandardized estimate (95% CI)	Standardized coefficient	***p-***value
**Direct effects**			
*Outcome model: SS (R*^*2*^ *= 0.186)*			
CAREi	0.205 (0.135; 0.275)	0.147	< 0.001
PD	0.456 (0.406; 0.507)	0.393	< 0.001
*Outcome model: Go to Doctor* ^a^ *(R*^*2*^ *= 0.104)*			
SS	0.353 (0.267; 0.438)	0.323	< 0.001
*Mediator model: PD (R*^*2*^ *= 0.08)*			
CAREi	0.109 (0.058; 0.161)	0.091	< 0.001
**Indirect effects (proportion mediated)**			
PD (8.2%)	0.050 (0.026; 0.074)	0.036	< 0.001
**Total effect**	0.607 (0.492; 0.722)	0.505	< 0.001

Abbreviations: *95% CI* 95% bias-corrected bootstrap confidence interval, *SS* Self-perceived stigmatization, *CAREi* Difficulties to adhere to preventive recommendations against COVID-19 infection without question 19, *PD* Psychological distress

^a^Answers flipped: higher score indicates that people would not go to a doctor in case of COVID-19 symptoms

In order to alleviate any concerns about the direction of the theorized main model, which is going from CARE to SS, the second sensitivity model consisted in reversing the order of the predictor and the outcome, thus replying to the question: does psychological distress mediates also the relationship between stigmatization and the use of preventive measures against COVID-19 spread?

As can be seen in the Supplementary Table 6, and Supplementary Fig. 1, we found significant direct effect of SS on CARE (B = 0.132; *p <* 0.001), supporting the likely existence of a vicious circle between the self-perceived stigmatization and the access to health care and the use of preventive measures against COVID-19 infection, though not significant indirect effect through PD (B = 0.007; bootstrap 95% CI: − 0.007 to 0.021) suggesting a unidirectional path from CARE to SS through PD, which is likely attributable to the specificity of the latent class CARE as conceptualized in the questionnaire. The fit indices were indicating good fit: CFI = 0.985, TLI = 0.983, RMSEA = 0.045, SRMR = 0.065.

Finally, to investigate the impact of potential confounders, some complementary models were assessed. Given previous work on the same sample [31], we included as covariates the housing situation and the residence status, gathered beneath the latent variable that we called socio-demographic hardship (SDH). For constructing the SDH latent variable, the housing status was dichotomized (house yes/no), the residence status was set on a three-level scale (permanent/temporary/no documents), therefore a higher SDH score was indicating socio-demographic instability. The SDH dimension resulted strongly associated with both PD and SS (B = 0.350; *p <* 0.001; and B = 0.274; *p <* 0.001, respectively). The effect of PD on SS is confirmed (B = 0.338; *p <* 0.001), as well as the direct effect of CARE on SS (B = 0.097; *p* = 0.015), yet the indirect effect through PD became no significant (B = -0.009; bootstrap 95% CI: − 0.030 to 0.011).

**Fig. 3:**
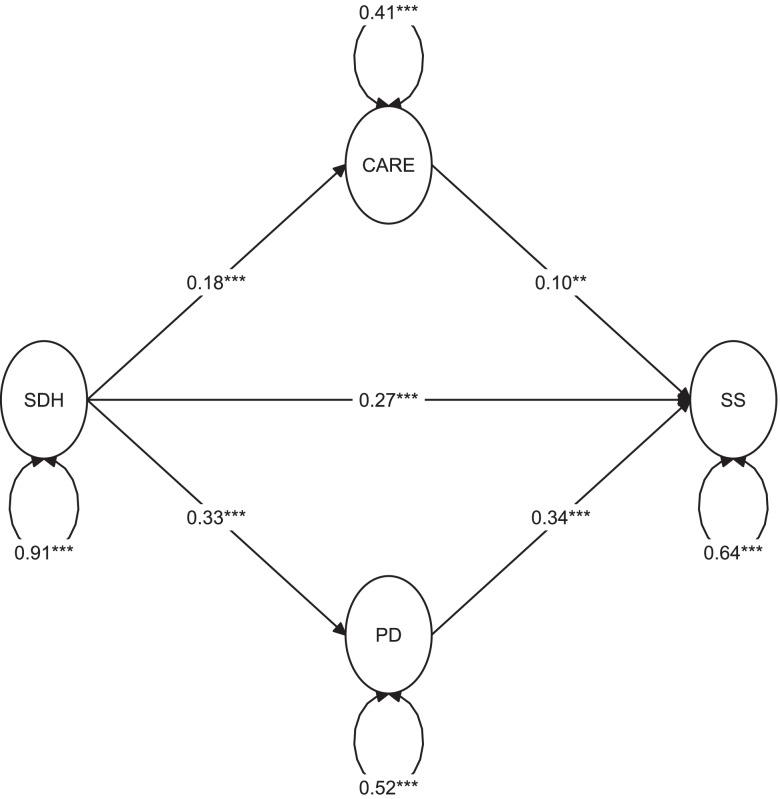
Parallel multiple mediator model in the full set. The estimates reported are the unstandardized regression coefficients. *p < 0.05; **p < 0.01; ***p < 0.001. Abbreviations: SDH: socio-demographic hardship; CARE: difficulties to adhere to preventive recommendations against COVID-19 infection; PD: psychological distress; SS: self-perceived stigmatization

From a theoretical perspective, it could be argued that SDH is a condition predisposing to increased difficulties in adhering to preventive measures against COVID-19. At the same time, being in disadvantaged living conditions may result in disengagement attitudes, based on not trusting the system or health care providers, expressed also with the refusal of governmental instructions of COVID-19 prevention. That, added to the fact that SDH is also strongly associated with both PD and SS, is suggesting a possible multiple mediator model where CARE and PD mediate the relationship between SDH and SS. As can be seen in Table 4 and Fig. 3, when testing that model, we could confirm that SDH exerts an effect on SS both directly (B = 0.265; *p <* 0.001) and indirectly through PD (B = 0.113; bootstrap 95% CI: 0.087 to 0.139) and CARE (B = 0.019; bootstrap 95% CI: 0.005 to 0.033). The fit indices were indicating a good fit: CFI = 0.983, TLI = 0.981, RMSEA = 0.044, SRMR = 0.066. Cronbach’s alpha for the latent variable SDH was 0.75.

**Table 4:** Results of parallel multiple mediator model in the full set

Regressions	Unstandardized estimate (95% CI)	Standardized coefficient	***p-***value
**Direct effects**			
*Outcome model: SS (R*^*2*^ *= 0.246)*			
SDH	0.265 (0.189; 0.341)	0.274	< 0.001
PD	0.342 (0.275; 0.409)	0.293	< 0.001
CARE	0.105 (0.028; 0.181)	0.075	0.007
*Mediator model: PD (R*^*2*^ *= 0.160)*			
SDH	0.330 (0.275; 0.385)	0.399	< 0.001
*Mediator model: CARE (R*^*2*^ *= 0.069)*			
SDH	0.183 (0.130; 0.235)	0.263	< 0.001
**Indirect effects (proportion mediated)**			
PD (28.5%)	0.113 (0.087; 0.139)	0.117	< 0.001
CARE (4.8%)	0.019 (0.005; 0.033)	0.020	0.007
**Total effect**	0.397 (0.327; 0.468)	0.411	< 0.001

Abbreviations: *95% CI* 95% bias-corrected bootstrap confidence interval, *SS* Self-perceived stigmatization, *SDH* Socio-demographic hardship, *CARE* Difficulties to adhere to preventive recommendations against COVID-19 infection, *PD* Psychological distress

## Discussion

This study set out to better describe the effect of the COVID-19 pandemic on refugees and other migrants in Europe. Specifically, we aimed to model the relationship between the difficulties to adhere to care and preventive measures against further spread of the COVID-19 infection, experience of self-perceived stigmatization, and psychological distress. We found that increased difficulties in following the preventive measures against COVID-19 are associated with greater psychological suffering and experience of discrimination. Moreover, psychological distress mediated the effect of preventive measures use on self-perceived stigmatization. The use of a single hold-out sample cross-validation strengthens our confidence in these findings. The relationship between the self-perceived stigmatization with both the adherence to preventive measures against COVID-19 infection and the psychological distress was robust after the adjustment for known conditions of social vulnerability, such as insecure housing situation and residence status.

Although the relationship between stigma and health is well known in vulnerable populations [21, 45, 46], this is the first study to confirm that access to care and to preventive measures influence the reporting of experience of discrimination, both directly and indirectly through its effect on psychological wellbeing.

In our models, psychological distress was defined as a condition of hyperarousal and anxious-depressive symptoms. Therefore, within the specific context of the COVID-19 pandemic, the relationship between facing barriers to access health care and preventive measures against COVID-19 infection (as indicated by higher CARE scores) and higher psychological distress (i.e., higher PD score) could reflect a fear of catching the virus [47]. In addition, lower levels of CARE might imply that people travel despite possible lockdown-measures or interact with others without wearing a face cover; those kinds of behaviors could be viewed as unsafe or irresponsible which might elicit discriminatory behaviors (such as refusal to provide service, harassment, and bullying), avoidant reactions in the host population [48] or the perception thereof [49]. That may be a plausible explanation for the relationship found between the lack of preventive measure utilization and the self-perceived stigmatization. With respect to the indirect effect of CARE on SS through PD, it could be hypothesized that the awareness to have difficulties in following public health measures against COVID-19 may create the expectation of being discriminated by the others, nurtured by negative psychological and emotional reactions [50].

According to the Theory of Social Stigma, for dealing with the experience of discrimination, people employ either active or passive coping strategies. The former reflects motivation for struggle and change, whereas the latter consists in elusive and disengagement attitudes [27, 28, 51]. Our findings and previous studies suggest that difficulties at following the recommendations against COVID-19 infection are higher among those with more unstable housing and residence status, which is likely due to no available income when the guidelines implied to buy products (mask, certain soap, disinfectant, etc.) [2, 52]. Also, access to information about COVID-19 preventive measures is a challenge in such conditions [9, 52]. That could be experienced by migrants as the failure of their migration project and social protection programs, leading them to lose trust in the host country. That may result also in passive coping strategies, involving elusive attitudes such as the rejection of state guidance for COVID-19 prevention, or reluctance to get vaccinated [15, 19, 53, 54]. From that perspective, the survey answers “No, because I’m not able” and “No, because I don’t want” to the question about the precautions use may be seen as two manifestations of the same psycho-social condition.

A vicious circle of discrimination and poor utilization of protection measures against COVID-19 can be created, and the finding of an association between the self-perceived stigmatization and the negative healthcare seeking attitude (i.e., the answer “No, I would not contact a doctor if I or someone close to me develop COVID-19 symptoms” to question 19) is supporting that hypothesis [55, 56].

### Limitations

This study should be interpreted in the light of its limitations. First, all participants were living in Europe, which may affect the generalizability of the findings to other continents. Second, the cross-sectional design limited the capability to address causality, and the implementation of a rigorous mediation path analysis approach has only partially bridged this limitation. In addition, there was not a control group of participants without migrant background. Future research should adopt prospective designs, particularly assessing the transition rates from non-clinical (self-reported) psychological distress to clinical psychopathology, and a control group to quantify the excess of vulnerability due to the migrant/refugee status. Third, due to the method of recruitment (which primarily took place online, via social media), and the COVID-19 restrictive measures, it was easier to reach a younger population with higher literacy and access to technical devices. It was as such also more difficult to contact populations from harder-to-reach groups, which are possibly the most impacted by the pandemic. Fourth, the survey was active over a seven-month period, meaning that respondents entered at different epidemiological stages, which varied both between countries and over time. Finally, the questionnaire was not validated before disseminating it, and there was a considerable number of missing entries that could not be imputed by the computation package. Therefore, the implementation of listwise deletion of the missing values limited the final sample size, moreover, future assessment of survey constructs, in terms of concurrency and convergency with other previously validated measures is desirable.

### Implication for research and practice

The study findings indicate that the COVID-19 pandemic severely impacts the most socially vulnerable fringes of society. Previous research works highlighted that disadvantaged populations, such as homeless people, migrants, refugees and applicants for international protection, experience difficult living conditions, previous experience of severe traumatic events and mental distress, that all are known to negatively impact mental health [(2, 57–60]). Although we could not quantify the excess of risk due to the migrant status for the absence of a control group, our findings complement that evidence, helping to identify intersectional factors of vulnerability in the migrant population and suggesting possible causal associations among them.

Future research should focus on protective factors and coping strategies that might mitigate the effect of the aforementioned stressors. Specifically, theoretical models, such as the Conservation of Resources Theory, could be inspiring or could even be validated in the context of the COVID-19 pandemic [61]. Furthermore, the use of qualitative data may be useful to better understand the nuances of the subjective experience of refugees and migrants and to let their voices heard. For instance, research using qualitative data found that during COVID-19 pandemics many of the migrants faced several challenges in accessing information in a language understandable to them and navigating constant streams of official news flows issuing instructions about which actions to take [52]. However, migrants have also found creative ways to address some of these challenges, often aided by digital tools [9].

It is important to reflect also on eventual differences across countries with respect to legal requirements in the COVID-19 response and in the healthcare entitlements, which may have had an impact on the adherence to recommendations against COVID-19. As well, there may be differences from country to country also in the level of social support, impacting on the housing situation, the residence status, and the access to information. Although this study was not aimed at investigating country-specific differences in the COVID-19 response, also due to the heterogeneous distribution of participants across countries, we expect further research could also help in elucidating the impact of different type of healthcare systems on the observed relations. Maybe the effect of the stressors is mitigated by the healthcare organization in terms of number of low thresholds services, stronger primary care, or with care providers well trained in intercultural skills. If that will be confirmed, effective political action needs to be taken to ensure access to health care services. An option may be to exploit mediating figures, such as the Community Health Workers, that have been confirmed to foster the access to care of migrants in the host country [62], and to reduce the social stigma by the host population [63]. Finally, our findings highlight the utmost need to include refugees and other migrants in COVID-19 vaccine campaigns, to reduce their risk of infection linked to their living conditions, and the negative impact on both mental health and experience of discrimination. That call for equitable access to COVID-19 vaccines is also in the WHO agenda [64].

## Conclusions

Overall, our findings contribute to understand the condition of refugees and other migrants in the context of COVID-19 pandemic. Those who have more difficulties in accessing health care and preventive measures against the infection experience deterioration of their mental health and increased discrimination by the host population. Refugees and other migrants with a more insecure housing situation and residence status are particularly vulnerable to these negative effects. This is a very relevant message for professionals and public policies in what highlighting from one side the specific risk of insecure housing in the impact of COVID-19 upon mental health and infection protection, and for another side the need to proper housing as a strategy to prevent both COVID-19 and mental distress.

## Supplementary Information

**Additional file 1.**

## Data Availability

The variance/covariance matrix is available in the supplementary. The codes for reproducing the main model are available at https://github.com/MattiaMarchi/ApartTogether

## Abbreviations

COVID-19: Coronavirus disease 2019
CARE: Difficulties to adhere to preventive recommendations against COVID-19 infection
SS: Self-perceived stigmatization
PD: Psychological distress
SEM: Structural equation modelling
DWLS: Diagonally weighted least squares estimator
95% CI: 95% confidence interval
BCa: Adjusted bootstrap percentile method
RMSEA: Root Means Square Error of Approximation
SRMR: Standardized Root Means Square Residual
TLI: Tucker–Lewis Index
CFI: Comparative Fit Index
SD: Standard deviation
B: Unstandardized regression coefficient beta
CAREi: Difficulties to adhere to preventive recommendations against COVID-19 infection without question 19
SDH: Socio-demographic hardship
