# Supplementary material for: Experience of discrimination during COVID-19 pandemic: the impact of public health measures and psychological distress among refugees and other migrants in Europe
Source: BMC Public Health. 2022 May 11;22:942. doi: 10.1186/s12889-022-13370-y (PMC9090600; doi:10.1186/s12889-022-13370-y)
Supplement: Supplementary file 1 — Additional file 1. [file 12889_2022_13370_MOESM1_ESM.docx]

**Supplementary Materials**

Table 1: variance/covariance matrix

|  | q26q1 | q26q2 | q26q3 | q26q4 | q26q5 | q26q6 | q17q1 | q17q2 | q17q3 | q17q4 | q17q5 | q19 | q29q1 | q29q2 | q29q3 | q29q4 | q29q5 | q29q6 | q29q7 | q29q8 | q29q9 | q29q10 | q29q11 |
| --- | --- | --- | --- | --- | --- | --- | --- | --- | --- | --- | --- | --- | --- | --- | --- | --- | --- | --- | --- | --- | --- | --- | --- |
| q26q1 | 0.42130255 | 0.34221027 | 0.33114242 | 0.36871511 | 0.35682984 | 0.31495359 | 0.30182205 | 0.330624 | 0.36074124 | 0.37172778 | 0.4556409 | 0.4287535 | 0.67530879 | 0.64266987 | 0.66682727 | 0.63961369 | 0.6544249 | 0.64651185 | 0.62549181 | 0.65269644 | 0.61838674 | 0.61552909 | 0.53688788 |
| q26q2 | 0.34221027 | 0.35520856 | 0.30994474 | 0.34051533 | 0.32961916 | 0.29567768 | 0.2760885 | 0.30586495 | 0.34224149 | 0.3424244 | 0.43577496 | 0.39685338 | 0.63409381 | 0.61108443 | 0.63044059 | 0.60558405 | 0.61411917 | 0.60772845 | 0.58772733 | 0.61178872 | 0.58852243 | 0.58318379 | 0.50561333 |
| q26q3 | 0.33114242 | 0.30994474 | 0.33597201 | 0.33512026 | 0.32792829 | 0.2969914 | 0.26915651 | 0.2949224 | 0.32696071 | 0.33571754 | 0.42270177 | 0.38578992 | 0.61624057 | 0.59423013 | 0.61202744 | 0.59072584 | 0.59077856 | 0.59088178 | 0.56888725 | 0.59250725 | 0.5750095 | 0.57008902 | 0.49338962 |
| q26q4 | 0.36871511 | 0.34051533 | 0.33512026 | 0.42408797 | 0.37572771 | 0.32166592 | 0.29021405 | 0.31622513 | 0.35504161 | 0.36270553 | 0.45453538 | 0.41795718 | 0.67184249 | 0.64749106 | 0.67222667 | 0.64017177 | 0.65362254 | 0.65097852 | 0.63623749 | 0.65021368 | 0.63384373 | 0.61956477 | 0.53934977 |
| q26q5 | 0.35682984 | 0.32961916 | 0.32792829 | 0.37572771 | 0.40077565 | 0.31775408 | 0.28191289 | 0.31250611 | 0.35135421 | 0.35490545 | 0.44851191 | 0.40857741 | 0.65784644 | 0.63671577 | 0.65584359 | 0.62967487 | 0.64317044 | 0.64341997 | 0.62284157 | 0.64233671 | 0.62243344 | 0.61136656 | 0.53207889 |
| q26q6 | 0.31495359 | 0.29567768 | 0.2969914 | 0.32166592 | 0.31775408 | 0.32086109 | 0.26574061 | 0.29025303 | 0.32636836 | 0.326769 | 0.41352145 | 0.37747763 | 0.61053334 | 0.59335359 | 0.61211423 | 0.58501891 | 0.58543114 | 0.5834988 | 0.56554086 | 0.58488245 | 0.56634475 | 0.56236378 | 0.49223962 |
| q17q1 | 0.30182205 | 0.2760885 | 0.26915651 | 0.29021405 | 0.28191289 | 0.26574061 | 0.85549569 | 0.62798549 | 0.62668112 | 0.60047146 | 0.68109612 | 0.54202644 | 0.6896255 | 0.68517036 | 0.69202483 | 0.65879534 | 0.66172974 | 0.64323717 | 0.62932122 | 0.65723771 | 0.63152458 | 0.6155793 | 0.52181968 |
| q17q2 | 0.330624 | 0.30586495 | 0.2949224 | 0.31622513 | 0.31250611 | 0.29025303 | 0.62798549 | 1.05652201 | 0.70239092 | 0.73365396 | 0.80717769 | 0.60883443 | 0.77624623 | 0.77640604 | 0.79687509 | 0.75315566 | 0.75542931 | 0.73229069 | 0.70821043 | 0.75427218 | 0.7126426 | 0.71137627 | 0.60046244 |
| q17q3 | 0.36074124 | 0.34224149 | 0.32696071 | 0.35504161 | 0.35135421 | 0.32636836 | 0.62668112 | 0.70239092 | 1.1779735 | 0.71050947 | 0.90082516 | 0.67519207 | 0.84545946 | 0.85426109 | 0.863326 | 0.80649622 | 0.82000621 | 0.78765588 | 0.77270316 | 0.83191237 | 0.77494289 | 0.77921469 | 0.67468364 |
| q17q4 | 0.37172778 | 0.3424244 | 0.33571754 | 0.36270553 | 0.35490545 | 0.326769 | 0.60047146 | 0.73365396 | 0.71050947 | 1.20221074 | 0.95537041 | 0.68413405 | 0.87533139 | 0.87172669 | 0.90644251 | 0.84630242 | 0.82657702 | 0.83423795 | 0.78901815 | 0.83713113 | 0.81872493 | 0.8021757 | 0.67446184 |
| q17q5 | 0.4556409 | 0.43577496 | 0.42270177 | 0.45453538 | 0.44851191 | 0.41352145 | 0.68109612 | 0.80717769 | 0.90082516 | 0.95537041 | 1.51861416 | 0.84561633 | 1.08439861 | 1.09154428 | 1.12887297 | 1.07307804 | 1.04113923 | 1.05238484 | 0.98688478 | 1.05991969 | 1.01935647 | 1.01193863 | 0.83739814 |
| q19 | 0.4287535 | 0.39685338 | 0.38578992 | 0.41795718 | 0.40857741 | 0.37747763 | 0.54202644 | 0.60883443 | 0.67519207 | 0.68413405 | 0.84561633 | 0.86704411 | 1.01014612 | 0.99296414 | 1.01571594 | 0.9688971 | 0.9349236 | 0.92527373 | 0.88566883 | 0.94758107 | 0.91216404 | 0.90610726 | 0.76879409 |
| q29q1 | 0.67530879 | 0.63409381 | 0.61624057 | 0.67184249 | 0.65784644 | 0.61053334 | 0.6896255 | 0.77624623 | 0.84545946 | 0.87533139 | 1.08439861 | 1.01014612 | 2.5426207 | 2.10097751 | 2.13009684 | 2.00398044 | 1.83955892 | 1.84036054 | 1.72956624 | 1.87338013 | 1.84346736 | 1.79888934 | 1.44237754 |
| q29q2 | 0.64266987 | 0.61108443 | 0.59423013 | 0.64749106 | 0.63671577 | 0.59335359 | 0.68517036 | 0.77640604 | 0.85426109 | 0.87172669 | 1.09154428 | 0.99296414 | 2.10097751 | 2.58744836 | 2.1679568 | 1.94400629 | 1.77401511 | 1.80367069 | 1.66481665 | 1.8210795 | 1.78534165 | 1.74989906 | 1.42026418 |
| q29q3 | 0.66682727 | 0.63044059 | 0.61202744 | 0.67222667 | 0.65584359 | 0.61211423 | 0.69202483 | 0.79687509 | 0.863326 | 0.90644251 | 1.12887297 | 1.01571594 | 2.13009684 | 2.1679568 | 2.60936244 | 1.97807119 | 1.85841336 | 1.90135504 | 1.75355857 | 1.88856887 | 1.85549408 | 1.82354955 | 1.46435001 |
| q29q4 | 0.63961369 | 0.60558405 | 0.59072584 | 0.64017177 | 0.62967487 | 0.58501891 | 0.65879534 | 0.75315566 | 0.80649622 | 0.84630242 | 1.07307804 | 0.9688971 | 2.00398044 | 1.94400629 | 1.97807119 | 2.43579263 | 1.80317263 | 1.8198312 | 1.71522151 | 1.84730851 | 1.79976863 | 1.7565105 | 1.42257981 |
| q29q5 | 0.6544249 | 0.61411917 | 0.59077856 | 0.65362254 | 0.64317044 | 0.58543114 | 0.66172974 | 0.75542931 | 0.82000621 | 0.82657702 | 1.04113923 | 0.9349236 | 1.83955892 | 1.77401511 | 1.85841336 | 1.80317263 | 2.21614158 | 1.83841823 | 1.75036501 | 1.91789942 | 1.77299769 | 1.74145655 | 1.41056077 |
| q29q6 | 0.64651185 | 0.60772845 | 0.59088178 | 0.65097852 | 0.64341997 | 0.5834988 | 0.64323717 | 0.73229069 | 0.78765588 | 0.83423795 | 1.05238484 | 0.92527373 | 1.84036054 | 1.80367069 | 1.90135504 | 1.8198312 | 1.83841823 | 2.26994107 | 1.74840414 | 1.87204354 | 1.77931976 | 1.7479388 | 1.41137144 |
| q29q7 | 0.62549181 | 0.58772733 | 0.56888725 | 0.63623749 | 0.62284157 | 0.56554086 | 0.62932122 | 0.70821043 | 0.77270316 | 0.78901815 | 0.98688478 | 0.88566883 | 1.72956624 | 1.66481665 | 1.75355857 | 1.71522151 | 1.75036501 | 1.74840414 | 2.01020785 | 1.80073686 | 1.69927129 | 1.66226314 | 1.36770041 |
| q29q8 | 0.65269644 | 0.61178872 | 0.59250725 | 0.65021368 | 0.64233671 | 0.58488245 | 0.65723771 | 0.75427218 | 0.83191237 | 0.83713113 | 1.05991969 | 0.94758107 | 1.87338013 | 1.8210795 | 1.88856887 | 1.84730851 | 1.91789942 | 1.87204354 | 1.80073686 | 2.30977483 | 1.82669059 | 1.80329721 | 1.46661518 |
| q29q9 | 0.61838674 | 0.58852243 | 0.5750095 | 0.63384373 | 0.62243344 | 0.56634475 | 0.63152458 | 0.7126426 | 0.77494289 | 0.81872493 | 1.01935647 | 0.91216404 | 1.84346736 | 1.78534165 | 1.85549408 | 1.79976863 | 1.77299769 | 1.77931976 | 1.69927129 | 1.82669059 | 2.20958189 | 1.75100804 | 1.40518965 |
| q29q10 | 0.61552909 | 0.58318379 | 0.57008902 | 0.61956477 | 0.61136656 | 0.56236378 | 0.6155793 | 0.71137627 | 0.77921469 | 0.8021757 | 1.01193863 | 0.90610726 | 1.79888934 | 1.74989906 | 1.82354955 | 1.7565105 | 1.74145655 | 1.7479388 | 1.66226314 | 1.80329721 | 1.75100804 | 2.20276299 | 1.41375006 |
| q29q11 | 0.53688788 | 0.50561333 | 0.49338962 | 0.53934977 | 0.53207889 | 0.49223962 | 0.52181968 | 0.60046244 | 0.67468364 | 0.67446184 | 0.83739814 | 0.76879409 | 1.44237754 | 1.42026418 | 1.46435001 | 1.42257981 | 1.41056077 | 1.41137144 | 1.36770041 | 1.46661518 | 1.40518965 | 1.41375006 | 1.45223858 |

Table 2: Detailed description of the study questionnaire answers

| **Variable (N=8297)** | **Frequency (%)/Mean (SD)** | **Missing rates** |
| --- | --- | --- |
| Country  Belgium  Germany  Norway  Hungary  Iceland  Netherlands  Finland  Kosovo  Lithuania  Luxembourg  Ireland  Malta  Albania  Poland  Romania  Russia  Denmark  Andorra  Serbia  Slovakia  Switzerland  Turkey  Ukraine  Armenia  Austria  France  Azerbaijan  Belarus  Greece  Bosnia and Herzegovina  Bulgaria  Italy  Spain  Croatia  Sweden  Cyprus  Czech Republic  United Kingdom  Estonia  Portugal  Georgia | 1894 (22.8%)  506 (6.1%)  91 (1.1%)  6 (0.1%)  3 (0.04%)  269 (3.2%)  45 (0.5%)  1 (0.01%)  12 (0.1%)  7 (0.08%)  183 (2.2%)  11 (0.1%)  86 (1%)  29 (0.3%)  32 (0.4%)  407 (4.9%)  681 (8.2%)  58 (0.7%)  4 (0.05%)  2 (0.02%)  41 (0.5%)  107 (1.3%)  5 (0.06%)  9 (0.1%)  76 (0.9%)  582 (7%)  8 (0.1%)  7 (0.08%)  311 (3.7%)  3 (0.04%)  3 (0.04%)  754 (9.1%)  406 (4.9%)  6 (0.1%)  443 (5.3%)  15 (0.2%)  13 (0.2%)  528 (6.4%)  4 (0.05%)  326 (3.9%)  1 (0.01%) | 323 (3.9%) |
| CARE:  What precautions are you taking to avoid the virus? (Always/Sometimes/Not able/Don’t want)  Hand washing  Physical distance  Cover nose and mouth  Avoid public transport  Avoid going out of my house  In case I or one of my family members develop COVID-19 symptoms, would I contact a doctor? (Yes/No) | 4437 (53.5%)/1083 (13%)/101 (1.2%)/48 (0.58%)  3819 (46%)/1440 (17.4%)/287 (3.5%)/54 (0.7%)  3054 (36.8%)/1586 (19.1%)/415 (5.0%)/516 (6.2%)  2985 (36%)/1644 (19.8%)/749 (9.0%)/190 (2.3%)  1586 (19.1%)/2503 (30.2%)/1034 (12.5%)/401 (4.8%)  5188 (62.5%)/377 (4.5%) | 2630 (31.7%)  2697 (32.5%)  2730 (32.9%)  2730 (32.9%)  2771 (33.4%)  2730 (32.9%) |
| PD:  Since the corona-crisis, have you been bothered by the following problems? (No/Seldom/Sometimes/Always)  Feeling depressed  Feeling like you worry too much  Feeling anxious  Feeling lonely  Feeling angry  Reminders of past traumatic experiences  Physical reactions to stress  Irritable  Feeling hopeless  Sleep problems  Using alcohol or drugs | 1154 (13.9%)/1293 (15.6%)/1565 (18.9%)/ 731 (8.8%)  780 (9.4%)/1344 (16.2%)/1595 (19.2%)/995 (12%)  954 (11.5%)/1380 (16.6%)/1615 (19.5%)/747 (9%)  1351 (16.3%)/1139 (13.7)/1434 (17.3)/746 (9%)  2086 (25.1%)/1076 (13%)/1131 (13.6%)/315 (3.8%)  1830 (22.1%)/1155 (13.9%)/1161 (14%)/406 (4.9%)  2556 (30.8%)/907 (10.9%)/900 (10.8%)/200 (2.4%)  1709 (20.6%)/1333 (16.1%)/1155 (13.9%)/354 (4.3%)  1854 (22.3%)/1133 (16.1%)/1096 (13.2%)/494 (6%)  1810 (21.8%)/1127 (13.6%)/1088 (13.1%)/545 (6.6%)  3577 (43.1%)/507 (6.1%)/350 (4.2%)/131 (1.6%) | 3551 (42.8%)  3584 (43.2%)  3601 (43.4%)  3626 (43.7%)  3692 (44.5%)  3742 (45.1%)  3734 (45%)  3742 (45.1%)  3717 (44.8%)  3725 (44.9%)  3734 (45%) |
| SS:  Since the corona-crisis, do you feel differently treated by others than before? (No/Yes)  Because of my origin  Treated with kindness  Others call me names  Others avoid me  Others are anxious about me  Police treat me unfairly | 4020 (48.5%)/865 (10.4%)  4277 (51.5%)/518 (6.2%)  4329 (52.2%)/419 (5.1%)  3891 (46.9%)/846 (10.2%)  3998 (48.2%)/728 (8.8%)  4366 (52.6%)/343 (4.1%) | 3410 (41.1%)  3501 (42.2%)  3551 (42.8%)  3559 (42.9%)  3568 (43%)  3584 (43.2%) |

Abbreviations: CARE: difficulties to adhere to preventative recommendations against COVID-19 infection; PD: psychological distress; SS: self-perceived stigmatization.

Table 3: CARE items endorsed by participants from each country

| **Country** | **n** | **Go to Doctor (%)** | **Handwashing (%)** | | | **Physical distance (%)** | | | **Cover nose and mouth (%)** | | | **Avoid public transport (%)** | | | **Avoid going out home (%)** | | |
| --- | --- | --- | --- | --- | --- | --- | --- | --- | --- | --- | --- | --- | --- | --- | --- | --- | --- |
|  |  | *No* | *Always* | *Sometimes* | *No* | *Always* | *Sometimes* | *No* | *Always* | *Sometimes* | *No* | *Always* | *Sometimes* | *No* | *Always* | *Sometimes* | *No* |
| ***Missing*** | 322 | 15 (8.0) | 162 (83.9) | 24 (12.4) | 7 (3.6) | 136 (72.0) | 37 (19.6) | 16 (8.5) | 110 (58.2) | 56 (29.6) | 23 (12.2) | 105 (55.6) | 47 (24.9) | 37 (19.6) | 50 (26.6) | 91 (48.4) | 47 (25.0) |
| ***Albania*** | 86 | 14 (31.1) | 23 (50.0) | 19 (41.3) | 4 (8.7) | 17 (37.8) | 17 (37.8) | 11 (24.4) | 16 (39.0) | 17 (41.5) | 8 (19.5) | 14 (32.6) | 12 (27.9) | 17 (39.5) | 4 (9.1) | 20 (45.5) | 20 (45.5) |
| ***Andorra*** | 58 | 3 (8.8) | 11 (33.3) | 18 (54.5) | 4 (12.1) | 10 (32.3) | 18 (58.1) | 3 (9.7) | 11 (34.4) | 17 (53.1) | 4 (12.5) | 11 (32.4) | 19 (55.9) | 4 (11.8) | 8 (26.7) | 17 (56.7) | 5 (16.7) |
| ***Armenia*** | 9 | 1 (12.5) | 5 (62.5) | 3 (37.5) | 0 (0.0) | 5 (62.5) | 3 (37.5) | 0 (0.0) | 6 (75.0) | 2 (25.0) | 0 (0.0) | 3 (42.9) | 4 (57.1) | 0 (0.0) | 2 (28.6) | 5 (71.4) | 0 (0.0) |
| ***Austria*** | 76 | 3 (4.3) | 60 (93.8) | 3 (4.7) | 1 (1.6) | 24 (38.7) | 35 (56.5) | 3 (4.8) | 54 (87.1) | 6 (9.7) | 2 (3.2) | 18 (29.5) | 35 (57.4) | 8 (13.1) | 10 (16.4) | 30 (49.2) | 21 (34.4) |
| ***Azerbaijan*** | 8 | 0 (0.0) | 2 (33.3) | 4 (66.7) | 0 (0.0) | 1 (20.0) | 4 (80.0) | 0 (0.0) | 1 (16.7) | 4 (66.7) | 1 (16.7) | 1 (20.0) | 4 (80.0) | 0 (0.0) | 0 (0.0) | 5 (100.0) | 0 (0.0) |
| ***Belarus*** | 7 | 0 (0.0) | 2 (28.6) | 3 (42.9) | 2 (28.6) | 2 (28.6) | 5 (71.4) | 0 (0.0) | 3 (42.9) | 3 (42.9) | 1 (14.3) | 2 (28.6) | 3 (42.9) | 2 (28.6) | 0 (0.0) | 6 (85.7) | 1 (14.3) |
| ***Belgium*** | 1894 | 44 (4.0) | 908 (80.7) | 196 (17.4) | 21 (1.9) | 907 (81.6) | 181 (16.3) | 24 (2.2) | 436 (39.5) | 418 (37.9) | 249 (22.6) | 728 (65.6) | 248 (22.3) | 134 (12.1) | 414 (37.4) | 549 (49.6) | 144 (13.0) |
| ***Bosnia and Herzegovina*** | 3 | 0 (0.0) | 0 (0.0) | 0 (0.0) | 1 (100.0) | 0 (0.0) | 0 (0.0) | 1 (100.0) | 0 (0.0) | 1 (100.0) | 0 (0.0) | 0 (0.0) | 1 (100.0) | 0 (0.0) | 0 (0.0) | 0 (0.0) | 1 (100.0) |
| ***Bulgaria*** | 3 | 0 (0.0) | 1 (50.0) | 1 (50.0) | 0 (0.0) | 1 (50.0) | 1 (50.0) | 0 (0.0) | 2 (100.0) | 0 (0.0) | 0 (0.0) | 0 (0.0) | 1 (50.0) | 1 (50.0) | 0 (0.0) | 1 (50.0) | 1 (50.0) |
| ***Croatia*** | 6 | 1 (20.0) | 4 (80.0) | 1 (20.0) | 0 (0.0) | 3 (60.0) | 2 (40.0) | 0 (0.0) | 4 (80.0) | 1 (20.0) | 0 (0.0) | 3 (60.0) | 2 (40.0) | 0 (0.0) | 2 (40.0) | 3 (60.0) | 0 (0.0) |
| ***Cyprus*** | 15 | 2 (14.3) | 14 (100.0) | 0 (0.0) | 0 (0.0) | 12 (92.3) | 1 (7.7) | 0 (0.0) | 14 (100.0) | 0 (0.0) | 0 (0.0) | 11 (78.6) | 1 (7.1) | 2 (14.3) | 7 (50.0) | 6 (42.9) | 1 (7.1) |
| ***Czech Republic*** | 13 | 1 (9.1) | 7 (63.6) | 4 (36.4) | 0 (0.0) | 3 (27.3) | 4 (36.4) | 4 (36.4) | 9 (81.8) | 2 (18.2) | 0 (0.0) | 3 (30.0) | 1 (10.0) | 6 (60.0) | 3 (27.3) | 2 (18.2) | 6 (54.5) |
| ***Denmark*** | 681 | 16 (3.2) | 414 (80.1) | 96 (18.6) | 7 (1.4) | 377 (72.8) | 128 (24.7) | 13 (2.5) | 126 (24.7) | 153 (30.0) | 231 (45.3) | 347 (67.5) | 116 (22.6) | 51 (9.9) | 128 (25.0) | 272 (53.0) | 113 (22.0) |
| ***Estonia*** | 4 | 1 (25.0) | 2 (50.0) | 2 (50.0) | 0 (0.0) | 3 (75.0) | 1 (25.0) | 0 (0.0) | 2 (50.0) | 2 (50.0) | 0 (0.0) | 2 (50.0) | 0 (0.0) | 2 (50.0) | 1 (25.0) | 0 (0.0) | 3 (75.0) |
| ***Finland*** | 45 | 3 (10.7) | 27 (93.1) | 1 (3.4) | 1 (3.4) | 23 (82.1) | 5 (17.9) | 0 (0.0) | 20 (71.4) | 6 (21.4) | 2 (7.1) | 15 (53.6) | 8 (28.6) | 5 (17.9) | 7 (25.0) | 9 (32.1) | 12 (42.9) |
| ***France*** | 582 | 28 (8.7) | 231 (71.5) | 76 (23.5) | 16 (5.0) | 205 (64.5) | 87 (27.4) | 26 (8.2) | 200 (63.1) | 85 (26.8) | 32 (10.1) | 151 (47.6) | 109 (34.4) | 57 (18.0) | 100 (31.7) | 138 (43.8) | 77 (24.4) |
| ***Georgia*** | 1 | 0 (0.0) | 1 (100.0) | 0 (0.0) | 0 (0.0) | 1 (100.0) | 0 (0.0) | 0 (0.0) | 1 (100.0) | 0 (0.0) | 0 (0.0) | 1 (100.0) | 0 (0.0) | 0 (0.0) | 0 (0.0) | 0 (0.0) | 1 (100.0) |
| ***Germany*** | 506 | 24 (6.6) | 297 (79.6) | 69 (18.5) | 7 (1.9) | 231 (62.3) | 123 (33.2) | 17 (4.6) | 246 (66.0) | 118 (31.6) | 9 (2.4) | 154 (41.7) | 138 (37.4) | 77 (20.9) | 82 (22.3) | 164 (44.7) | 121 (33.0) |
| ***Greece*** | 311 | 15 (9.1) | 114 (65.1) | 43 (24.6) | 18 (10.3) | 78 (45.9) | 63 (37.1) | 29 (17.1) | 76 (45.2) | 59 (35.1) | 33 (19.6) | 76 (45.2) | 57 (33.9) | 35 (20.8) | 44 (26.3) | 68 (40.7) | 55 (32.9) |
| ***Hungary*** | 6 | 2 (40.0) | 5 (100.0) | 0 (0.0) | 0 (0.0) | 2 (40.0) | 2 (40.0) | 1 (20.0) | 4 (80.0) | 1 (20.0) | 0 (0.0) | 1 (20.0) | 4 (80.0) | 0 (0.0) | 1 (20.0) | 2 (40.0) | 2 (40.0) |
| ***Iceland*** | 3 | 1 (50.0) | 2 (100.0) | 0 (0.0) | 0 (0.0) | 1 (100.0) | 0 (0.0) | 0 (0.0) | 1 (100.0) | 0 (0.0) | 0 (0.0) | 1 (100.0) | 0 (0.0) | 0 (0.0) | 0 (0.0) | 0 (0.0) | 1 (100.0) |
| ***Ireland*** | 183 | 7 (4.7) | 126 (82.9) | 19 (12.5) | 7 (4.6) | 97 (65.1) | 36 (24.2) | 16 (10.7) | 77 (51.3) | 43 (28.7) | 30 (20.0) | 91 (60.7) | 30 (20.0) | 29 (19.3) | 31 (20.5) | 69 (45.7) | 51 (33.8) |
| ***Italy*** | 754 | 29 (5.6) | 405 (77.6) | 103 (19.7) | 14 (2.7) | 338 (65.8) | 133 (25.9) | 43 (8.4) | 429 (83.0) | 72 (13.9) | 16 (3.1) | 220 (43.1) | 173 (33.9) | 117 (22.9) | 150 (29.2) | 218 (42.4) | 146 (28.4) |
| ***Kosovo*** | 1 | 0 (0.0) | 1 (100.0) | 0 (0.0) | 0 (0.0) | 1 (100.0) | 0 (0.0) | 0 (0.0) | 1 (100.0) | 0 (0.0) | 0 (0.0) | 0 (0.0) | 1 (100.0) | 0 (0.0) | 0 (0.0) | 0 (0.0) | 1 (100.0) |
| ***Lithuania*** | 12 | 2 (16.7) | 7 (63.6) | 4 (36.4) | 0 (0.0) | 8 (72.7) | 3 (27.3) | 0 (0.0) | 9 (81.8) | 2 (18.2) | 0 (0.0) | 5 (50.0) | 5 (50.0) | 0 (0.0) | 4 (36.4) | 6 (54.5) | 1 (9.1) |
| ***Luxembourg*** | 7 | 0 (0.0) | 4 (57.1) | 3 (42.9) | 0 (0.0) | 5 (71.4) | 2 (28.6) | 0 (0.0) | 7 (100.0) | 0 (0.0) | 0 (0.0) | 1 (14.3) | 1 (14.3) | 5 (71.4) | 0 (0.0) | 6 (85.7) | 1 (14.3) |
| ***Malta*** | 11 | 0 (0.0) | 7 (87.5) | 0 (0.0) | 1 (12.5) | 4 (50.0) | 2 (25.0) | 2 (25.0) | 7 (87.5) | 1 (12.5) | 0 (0.0) | 2 (25.0) | 3 (37.5) | 3 (37.5) | 0 (0.0) | 5 (62.5) | 3 (37.5) |
| ***Netherlands*** | 269 | 11 (7.0) | 113 (71.1) | 38 (23.9) | 8 (5.0) | 105 (65.6) | 33 (20.6) | 22 (13.8) | 34 (21.7) | 54 (34.4) | 69 (43.9) | 85 (53.5) | 50 (31.4) | 24 (15.1) | 38 (23.6) | 87 (54.0) | 36 (22.4) |
| ***Norway*** | 91 | 2 (3.5) | 56 (90.3) | 6 (9.7) | 0 (0.0) | 51 (82.3) | 8 (12.9) | 3 (4.8) | 22 (36.7) | 20 (33.3) | 18 (30.0) | 24 (40.0) | 25 (41.7) | 11 (18.3) | 17 (29.8) | 24 (42.1) | 16 (28.1) |
| ***Poland*** | 29 | 3 (11.5) | 20 (76.9) | 4 (15.4) | 2 (7.7) | 19 (73.1) | 6 (23.1) | 1 (3.8) | 22 (84.6) | 3 (11.5) | 1 (3.8) | 8 (32.0) | 12 (48.0) | 5 (20.0) | 7 (26.9) | 8 (30.8) | 11 (42.3) |
| ***Portugal*** | 326 | 12 (6.0) | 175 (84.5) | 32 (15.5) | 0 (0.0) | 149 (73.0) | 50 (24.5) | 5 (2.5) | 153 (74.6) | 45 (22.0) | 7 (3.4) | 132 (64.1) | 46 (22.3) | 28 (13.6) | 73 (35.8) | 93 (45.6) | 38 (18.6) |
| ***Romania*** | 32 | 0 (0.0) | 22 (75.9) | 7 (24.1) | 0 (0.0) | 17 (58.6) | 6 (20.7) | 6 (20.7) | 19 (65.5) | 10 (34.5) | 0 (0.0) | 13 (44.8) | 7 (24.1) | 9 (31.0) | 5 (17.2) | 15 (51.7) | 9 (31.0) |
| ***Russia*** | 407 | 77 (19.8) | 286 (73.0) | 100 (25.5) | 6 (1.5) | 213 (54.6) | 138 (35.4) | 39 (10.0) | 258 (65.8) | 109 (27.8) | 25 (6.4) | 160 (41.6) | 135 (35.1) | 90 (23.4) | 64 (18.0) | 88 (24.7) | 204 (57.3) |
| ***Serbia*** | 4 | 0 (0.0) | 2 (50.0) | 2 (50.0) | 0 (0.0) | 3 (75.0) | 1 (25.0) | 0 (0.0) | 3 (75.0) | 1 (25.0) | 0 (0.0) | 3 (75.0) | 1 (25.0) | 0 (0.0) | 3 (75.0) | 1 (25.0) | 0 (0.0) |
| ***Slovakia*** | 2 | 0 (0.0) | 2 (100.0) | 0 (0.0) | 0 (0.0) | 0 (0.0) | 2 (100.0) | 0 (0.0) | 0 (0.0) | 2 (100.0) | 0 (0.0) | 2 (100.0) | 0 (0.0) | 0 (0.0) | 0 (0.0) | 1 (50.0) | 1 (50.0) |
| ***Spain*** | 406 | 10 (3.6) | 216 (77.1) | 60 (21.4) | 4 (1.4) | 201 (72.3) | 67 (24.1) | 10 (3.6) | 206 (73.8) | 61 (21.9) | 12 (4.3) | 176 (63.8) | 69 (25.0) | 31 (11.2) | 127 (45.7) | 101 (36.3) | 50 (18.0) |
| ***Sweden*** | 443 | 21 (6.5) | 270 (83.1) | 45 (13.8) | 10 (3.1) | 221 (70.2) | 82 (26.0) | 12 (3.8) | 77 (25.0) | 104 (33.8) | 127 (41.2) | 160 (50.6) | 109 (34.5) | 47 (14.9) | 62 (20.0) | 159 (51.3) | 89 (28.7) |
| ***Switzerland*** | 41 | 0 (0.0) | 24 (82.8) | 4 (13.8) | 1 (3.4) | 19 (65.5) | 9 (31.0) | 1 (3.4) | 21 (75.0) | 6 (21.4) | 1 (3.6) | 8 (28.6) | 11 (39.3) | 9 (32.1) | 8 (29.6) | 6 (22.2) | 13 (48.1) |
| ***Turkey*** | 107 | 10 (11.2) | 73 (79.3) | 19 (20.7) | 0 (0.0) | 64 (69.6) | 21 (22.8) | 7 (7.6) | 71 (77.2) | 15 (16.3) | 6 (6.5) | 42 (45.7) | 22 (23.9) | 28 (30.4) | 34 (37.0) | 35 (38.0) | 23 (25.0) |
| ***United Kingdom*** | 528 | 19 (4.7) | 333 (80.4) | 74 (17.9) | 7 (1.7) | 260 (63.6) | 123 (30.1) | 26 (6.4) | 295 (73.0) | 85 (21.0) | 24 (5.9) | 206 (51.2) | 133 (33.1) | 63 (15.7) | 100 (25.0) | 193 (48.2) | 107 (26.8) |
| ***Ukraine*** | 5 | 0 (0.0) | 3 (100.0) | 0 (0.0) | 0 (0.0) | 2 (66.7) | 1 (33.3) | 0 (0.0) | 1 (33.3) | 2 (66.7) | 0 (0.0) | 0 (0.0) | 1 (33.3) | 2 (66.7) | 0 (0.0) | 0 (0.0) | 3 (100.0) |
| **Median %** |  | 5.8 | 79.5 | 18.6 | 1.4 | 65.6 | 30.0 | 3.7 | 72.2 | 25.0 | 3.3 | 45.0 | 33.6 | 16.8 | 25.0 | 45.1 | 29.9 |
| **p-value** |  | <0.001 | <0.001 | | | <0.001 | | | <0.001 | | | <0.001 | | | <0.001 | | |

Table 4: Latent variable estimation in the full set

| **Latent variable** | **Observed variable** | **Unstandardized estimate (95% CI)** | **Standardized coefficient** | **p-value** |
| --- | --- | --- | --- | --- |
| *CARE* | *What precautions are you taking to avoid transmitting the coronavirus?* |  |  |  |
|  | Handwashing | Ref. 1.00 (1.00; 1.00) | 0.66 | NA |
|  | Physical distance | 1.18 (1.08; 1.28) | 0.78 | <0.001 |
|  | Cover nose and mouth | 0.44 (0.37; 0.51) | 0.29 | <0.001 |
|  | Avoid public transport | 0.98 (0.90; 1.07) | 0.65 | <0.001 |
|  | Avoid going out home | 0.88 (0.81; 0.96) | 0.59 | <0.001 |
|  | *In case I or one of my family members develops symptoms, I would contact a doctor or health care provider?* | 0.68 (0.56; 0.80) | 0.45 | <0.001 |
| *PD* | *Since the coronavirus and the corona measures, have you been bothered by the following problems?* |  |  |  |
|  | Feeling depressed | Ref. 1.00 (1.00; 1.00) | 0.79 | NA |
|  | Feeling anxious | 0.95 (0.93; 0.98) | 0.75 | <0.001 |
|  | Feeling lonely | 0.93 (0.90; 0.95) | 0.73 | <0.001 |
|  | Feeling angry | 1.01 (0.98; 1.03) | 0.79 | <0.001 |
|  | Reminders of past traumatic experiences | 0.92 (0.89; 0.94) | 0.72 | <0.001 |
|  | Physical reactions to stress | 0.89 (0.86; 0.92) | 0.70 | <0.001 |
|  | Irritable | 0.95 (0.93; 0.98) | 0.75 | <0.001 |
|  | Feeling hopeless | 0.92 (0.90; 0.95) | 0.73 | <0.001 |
|  | Sleep problems | 0.90 (0.87; 0.93) | 0.71 | <0.001 |
|  | Using alcohol or drugs | 0.47 (0.42; 0.53) | 0.37 | <0.001 |
| *SS* | *Since the corona-crisis, do you feel differently treated by others than before?* |  |  |  |
|  | Because of my origin | Ref. 1.00 (1.00; 1.00) | 0.92 | NA |
|  | Treated with kindness | 0.93 (0.90; 0.96) | 0.85 | <0.001 |
|  | Others call me names | 0.98 (0.95; 1.02) | 0.90 | <0.001 |
|  | Others avoid me | 0.98 (0.95; 1.01) | 0.90 | <0.001 |
|  | Others are anxious about me | 0.98 (0.96; 1.01) | 0.90 | <0.001 |
|  | Police treat me unfairly | 0.89 (0.84; 0.93) | 0.81 | <0.001 |

Abbreviations: CARE: difficulties to adhere to preventative recommendations against COVID-19 infection; PD: psychological distress; SS: self-perceived stigmatization.

Table 5: results of the sensitivity analysis on the full set of data

| **Path** | **Unstandardized estimate** | **p-value** | **p-value changed** | **Mean sensitivity estimate (min; max)** | **Mean % change** |
| --- | --- | --- | --- | --- | --- |
| PD ~ CARE | 0.098 | <0.001 | 0.050 | 0.0153 (-1.07; 1.21) | 84.5% |
| SS ~ CARE | 0.154 | <0.001 | 0.077 | 0.249 (-0.761; 1.10) | 61.7% |
| SS ~ PD | 0.392 | <0.001 | NA | 0.595 (-0.406; 1.25) | 51.8% |

Abbreviations: SS: self-perceived stigmatization; CARE: difficulties to adhere to preventative recommendations against COVID-19 infection; PD: psychological distress.

Mean % change is the absolute value of the difference between Unstandardized estimate and Mean sensitivity estimate divided by Unstandardized estimate. NA indicates that there is no change in the p-value for any of the tested phantom variable path coefficients.

Table 6: Sensitivity mediation model on the full set: predictor and outcome reversed

| **Regressions** | **Unstandardized estimate (95% CI)** | **Standardized coefficient** | **p-value** |
| --- | --- | --- | --- |
| **Direct effects** |  |  |  |
| *Outcome model: CARE (R^2^=0.166)* |  |  |  |
| SS | 0.132 (0.088; 0.177) | 0.183 | <0.001 |
| PD | 0.020 (-0.021; 0.062) | 0.024 | 0.335 |
|  |  |  |  |
| *Mediator model: PD (R^2^=0.038)* |  |  |  |
| SS | 0.350 (0.312; 0.388) | 0.407 | <0.001 |
|  |  |  |  |
| **Indirect effect (proportion mediated)** |  |  |  |
| PD (5.0%) | 0.007 (-0.007; 0.021) | 0.010 | 0.333 |
|  |  |  |  |
| **Total effect** | 0.139 (0.100; 0.179) | 0.192 | <0.001 |

Abbreviations: 95% CI: 95% bias-corrected bootstrap confidence interval; CARE: difficulties to adhere to preventive recommendations against COVID-19 infection; SS: self-perceived stigmatization; PD: psychological distress.


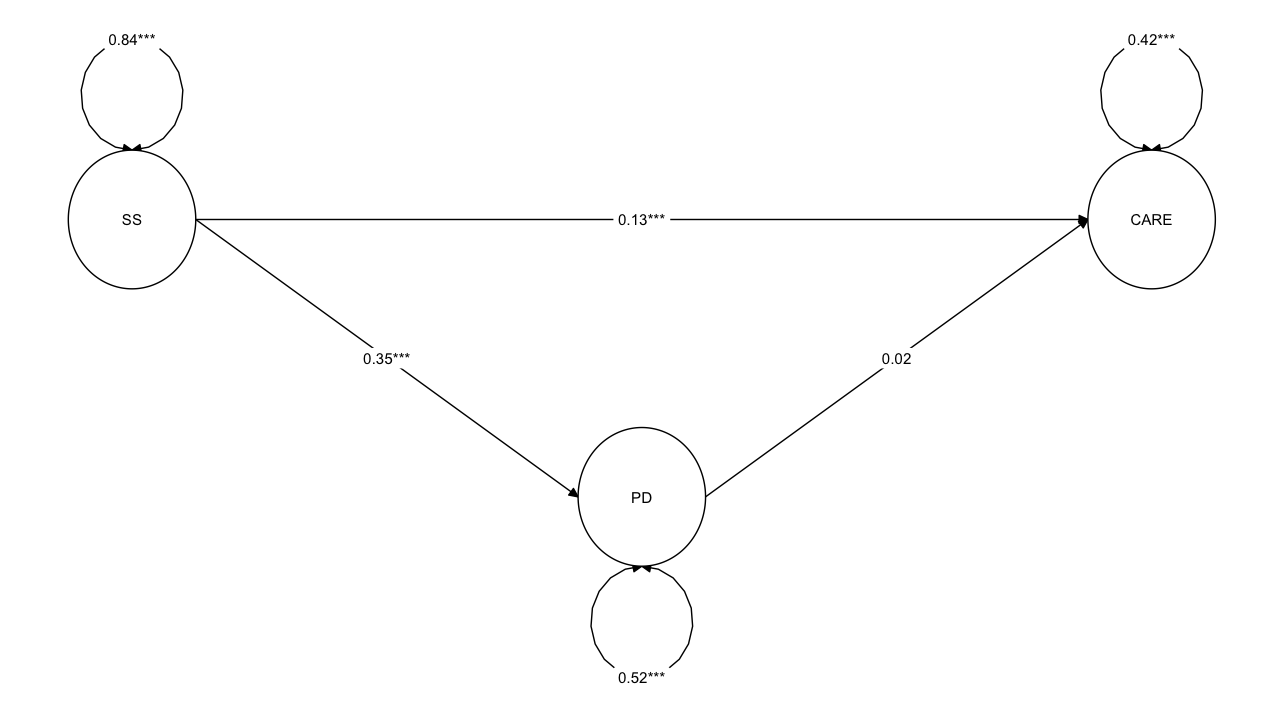


Figure 1: Sensitivity mediation model on the full set: predictor and outcome reversed.

Legend: The estimates reported are the unstandardized regression coefficients. *p<0.05; **p<0.01; ***p<0.001.

Abbreviations: SS: self-perceived stigmatization; PD: psychological distress; CARE: difficulties to adhere to preventive recommendations against COVID-19 infection.
